# 856. CT-proET-1 and sFlt-1 as Predictors of Clinical Outcomes in a COVID-19 US Cohort

**DOI:** 10.1093/ofid/ofad500.901

**Published:** 2023-11-27

**Authors:** Johnny Atallah, Natalie Atallah, Vahe Panossian, Hailey Warren, Damian Inlall, Andrej Schwabe, Sascha Johannes, Jan Wiemer, Michael Mansour

**Affiliations:** Massachusetts General Hospital, UConn Health, Hartford, Connecticut; Massachusetts General Hospital, Boston, Massachusetts; Massachusetts General Hospital, Boston, Massachusetts; Massachusetts General Hospital, Boston, Massachusetts; Thermofisher Scientific, Boston, Massachusetts; Thermofisher Scientific, Boston, Massachusetts; Thermofisher Scientific, Boston, Massachusetts; Thermofisher Scientific, Boston, Massachusetts; Massachusetts General Hospital, Boston, Massachusetts

## Abstract

**Background:**

Several novel biomarkers have been studied in predicting clinical outcomes in patients with COVID-19. Our study aimed to study C-terminal proendothelin-1 (CT-proET-1) and Soluble fms-like tyrosine kinase-1 (sFlt-1) as predictors of clinical outcomes in a COVID-19 patient cohort from a US-based multicenter trial.

**Methods:**

Data from the Boston Area COVID-19 Consortium (BACC) Bay Tocilizumab Trial was used in this study. Patients with biomarker determinations, and not admitted to the intensive care unit (ICU) on admission, were included. Median concentrations of CT-proET-1 (83.6 pmol/L) and sFlt-1 (81.3 pg/mL) were used as cutoffs in predicting clinical outcomes.

**Results:**

Out of 169 patients, 10.7% mechanically ventilated or dead within 28 days of admission. CT-proET1 concentrations in patients who were mechanically ventilated or dead within 28 days were significantly higher than those who were not (median 119.6 vs 79.1 pmol/L, p=0.001), and higher in patients who were admitted to the ICU during hospitalization compared to those who were not (median 114.5 vs 80.6 pmol/L, p=0.005). Significantly higher sFlt-1 concentrations were seen in patients who were admitted to the ICU compared to those who were not (median 96.0 vs 80.7 pg/mL, p=0.041).

The sensitivity, specificity, PPV and NPV of CT-proET-1 with cutoff 83.6 pmol/L in predicting mechanical ventilation or death were 83%, 54%, 18%, and 96%, respectively, with an AUC of 0.75.

As for the sFlt-1 cutoff of 81.3 pg/mL in predicting mechanical ventilation or death, the sensitivity, specificity, PPV and NPV were 61%, 54%, 14%, and 92%, with an AUC of 0.59.

**Conclusion:**

CT-proET-1 and sFlt-1 function as valuable novel biomarkers in predicting clinical outcomes in COVID-19, with lower levels predicting better outcomes. Further studies are needed to better understand their role in other upper respiratory viral illnesses.

**Disclosures:**

**Michael Mansour, MD, PhD**, Thermofisher: Grant/Research Support

